# Identification and in silico characterization of a novel p.P208PfsX1 mutation in V-ATPase a3 subunit associated with autosomal recessive osteopetrosis in a Pakistani family

**DOI:** 10.1186/s12881-017-0506-4

**Published:** 2017-12-13

**Authors:** Muhammad Ajmal, Asif Mir, Sughra Wahid, Chiea Chuen Khor, Jia Nee Foo, Saima Siddiqi, Mehran Kauser, Salman Akbar Malik, Muhammad Nasir

**Affiliations:** 1 Institute of Biomedical and Genetic Engineering, 24-Mauve area, G-9/1, Islamabad, 44000 Pakistan; 2 Department of Biotechnology, International Islamic university, Islamabad, Pakistan; 3 KRL General Hospital, Pediatric Department 24-Mauve area, G-9/1, Islamabad, 44000 Pakistan; 4 Human Genetics, Genome Institute of Singapore, A*STAR, Singapore, Singapore; 5 Department of Biochemistry, Yong Loo Lin School of Medicine, National University of Singapore, Singapore, Singapore; 6 Lee Kong Chian School of Medicine, Nanyang Technological University, Singapore, Singapore; 7 Department of Biochemistry, Quaid-i-Azam University, Islamabad, 44000 Pakistan

**Keywords:** Infantile malignant osteopetrosis, Human *TCIRG1* gene, V-ATPase, V0 domain, a3 subunit

## Abstract

**Background:**

Osteopetrosis is a rare inherited bone disorder mainly described as an increased bone density caused by defective osteoclastic bone resorption. To date, genetic variants of eleven genes have been reported so far to be associated with different types of osteopetrosis. However, malignant infantile osteopetrosis, a lethal form of the disease, is mostly (50%) caused by mutation(s) in *TCIRG1* gene. In this study, we investigated a consanguineous Pakistani family clinically and genetically to elucidate underlying molecular basis of the infantile osteopetrosis.

**Methods:**

DNA samples from five family members were subjected to SNP-array based whole genome homozygosity mapping. Data was analyzed and potentially pathogenic mutation was identified by Sanger sequencing of two affected as well as three phenotypically healthy individuals in the family. The significance of identified pathogenic variation and its impact on protein structure and function was studied using various bioinformatics tools.

**Results:**

DNA samples from five family members were subjected to genome-wide SNP array genotyping and homozygosity mapping which identified ~4 Mb region on chr11 harboring the *TCIRG1* gene. Sanger sequencing unveiled a novel homozygous deletion c. 624delC in exon 6 of the *TCIRG1* gene encodes a3 subunit of V-ATPase complex. The identified deletion resulted in a frame shift producing a truncated protein of 208 aa. In silico analysis of premature termination of the a3 subunit of V-ATPase complex revealed deleterious effects on the protein structure, predicting impaired or complete loss of V-ATPase function causing infantile osteopetrosis.

**Conclusions:**

Since a3 subunit of V-ATPase complex plays a crucial role in bone resorption process, structurally abnormal a3 subunit might have adversely affected bone resorption process, leading to infantile osteopetrosis in Pakistani family. Therefore, the present study not only expands the genotypic spectrum of osteopetrosis but also improve understandings of the role of V-ATPase a3 subunit in bone resorption process. Moreover, our findings should help in genetic counseling and provide further insight into the disease pathogenesis and potential targeted therapy.

**Electronic supplementary material:**

The online version of this article (10.1186/s12881-017-0506-4) contains supplementary material, which is available to authorized users.

## Background

Osteopetrosis (OPT) is a rare genetic disorder of bones, first reported in early 1904 [1]. Abnormally dense bone mass is a characteristic feature of osteopetrosis caused by a defective Osteoclastic bone resorption [2]. The condition lead to bone fragility and an increased vulnerability to fractures [3]. Four sub-types of OPT are described including infantile malignant OPT (OPTB1), adult/ benign OPT, intermediate OPT and carbon anhydrase type II deficiency syndrome [4]. However, Syndromic forms of osteopetrosis such as carbonic anhydrase 2 deficiency are not considered as classic autosomal recessive osteopetrosis (ARO) due to the mild presentation of the bone defect in affected patients [2]. Modes of inheritance further split osteopetrosis into autosomal dominant and autosomal recessive categories [5]. However, infantile malignant OPT, also known as autosomal recessive osteopetrosis type 1, remains the most common and best identified subtypes with an average disease frequency of 1/ 250,000 (1/200,000–1/300,000). The highest frequencies of infantile malignant OPT has been reported in populations of Costa-Rica (3.4/100,000) in Central American and Chuvash (1/3879 newborns) in Russia owing to a founder effect, high parental consanguinity or geographic isolation [2, 6].

Infantile malignant OPT is associated with intermediate and severe clinical phenotypes associated with reduced or complete loss of bone resorption capability of osteoclasts [7]. Impaired osteoclastic bone resorption leads to retention of old bones, hence an increase in bone density and obstruction of internal cavities containing vital tissues/organs such as bone marrow, nervous system etc. Distinctive symptoms include short stature, bone deformities and pathological fractures that may accompanied by severe hematological and neural failures [8]. In comparison with autosomal dominant benign OPT that occurs in late ages and with less severe clinical consequences, autosomal recessive infantile malignant OPT involves an early onset and lethal outcome [9]. The mortality rate of autosomal recessive OPT is significantly high during the first 2 years of life. Bone marrow failure, visual impairment and profuse infections before the age of 3 months are the major contributing factors to death [7].

Bone resorption depends on osteoclast secretion of hydrogen ions (H+), chloride ions (Cl−) and matrix-degrading protease, cathepsin K, into the resorption lacunae. The process mainly involves the breakdown of crystalline hydroxyapatite and collagen-rich organic bone matrix. The breakdown of both components requires acidic conditions, because hydroxyapatite is solubilized in acidic solution and the collagen-degrading protease, cathepsin K, is optimally active at acidic pH. Therefore, the bone surface acidic microenvironment beneath the ruffled border is a fundamental and crucial step for bone resorption. A continuous expulsion of H+ and Cl − through combined actions of V-ATPase proton pump and chloride channel in the ruffled border of osteoclasts serves to achieve this acidic microenvironment in resorptive lacunae. The degraded bone matrix is endocytosed from the resorption lacunae and finally released into the extracellular environment [10, 11]. The proton pump function of multi-subunit V-ATPase is tightly regulated by different genes whose pathogenic variants are frequently reported in impaired proton pump regulation. To date, 11 osteopetrosis genes have been identified including 7 genes associated with ARO; TCIRG1, CLCN7, OSTM1, SNX10, TNFSFR11A, TNFSF11 and PLEKHM1 [2, 5]. Four of the known ARO genes, *TCIRG1*, *CLCN7, OSTM1* and *SNX10* are significantly expressed in ruffled border of mature osteoclasts and are largely known for their involvement in autosomal recessive osteopetrosis. Specific proteins encoded by these genes are part of the osteoclast-specific enzymatic system that significantly contributes in bone dissolution process. The *TCIRG1* encodes a3 subunit of proton pump, is the most frequently mutated gene responsible for >50% of ARO cases. The *CLCN7* gene encodes chloride channel (CLC-7) to provide electro-neutrality during acidification process, account for approximately 13–16% of ARO cases. Mutations in a recently cloned *OSTM1* gene which encode transmembrane protein 1, lead to severe phenotypes and account for 2–6% ARO cases. Whereas, *SNX10* gene encodes sorting nexin 10, is accounting for almost 4% ARO cases. Overall, approximately >70% malignant infantile osteopetrosis cases emerge from mutations in these four genes. The remaining 3 gene are less frequently described in ARO patients with TNFSFR11A in <1–4% of cases, TNFSF11 in <1–3% of cases and only 2 cases of PLEKHM1 have been reported in the literature so far [2].

Though the incidence of disorder is worldwide without any gender specificity, it is frequently reported in ethnic groups with increased consanguinity [7]. Pakistan is also a country where consanguinity is being practiced over hundreds of years by highly conserved ethnic groups [12]. As a result, variety of inherited bone disorders including osteopetrosis can easily be identified. Unfortunately, no significant work has been carried out so far to identify molecular basis of osteopetrosis in Pakistani population. In the present study, a multi-generational consanguineous Pakistani family suffering from infantile osteopetrosis was clinically and genetically characterized. Genetic screening revealed a novel homozygous deletion mutation in V-ATPase a3 subunit associated with autosomal recessive osteopetrosis in this Pakistani family.

## Methods

### Subjects

**Fig. 1:**
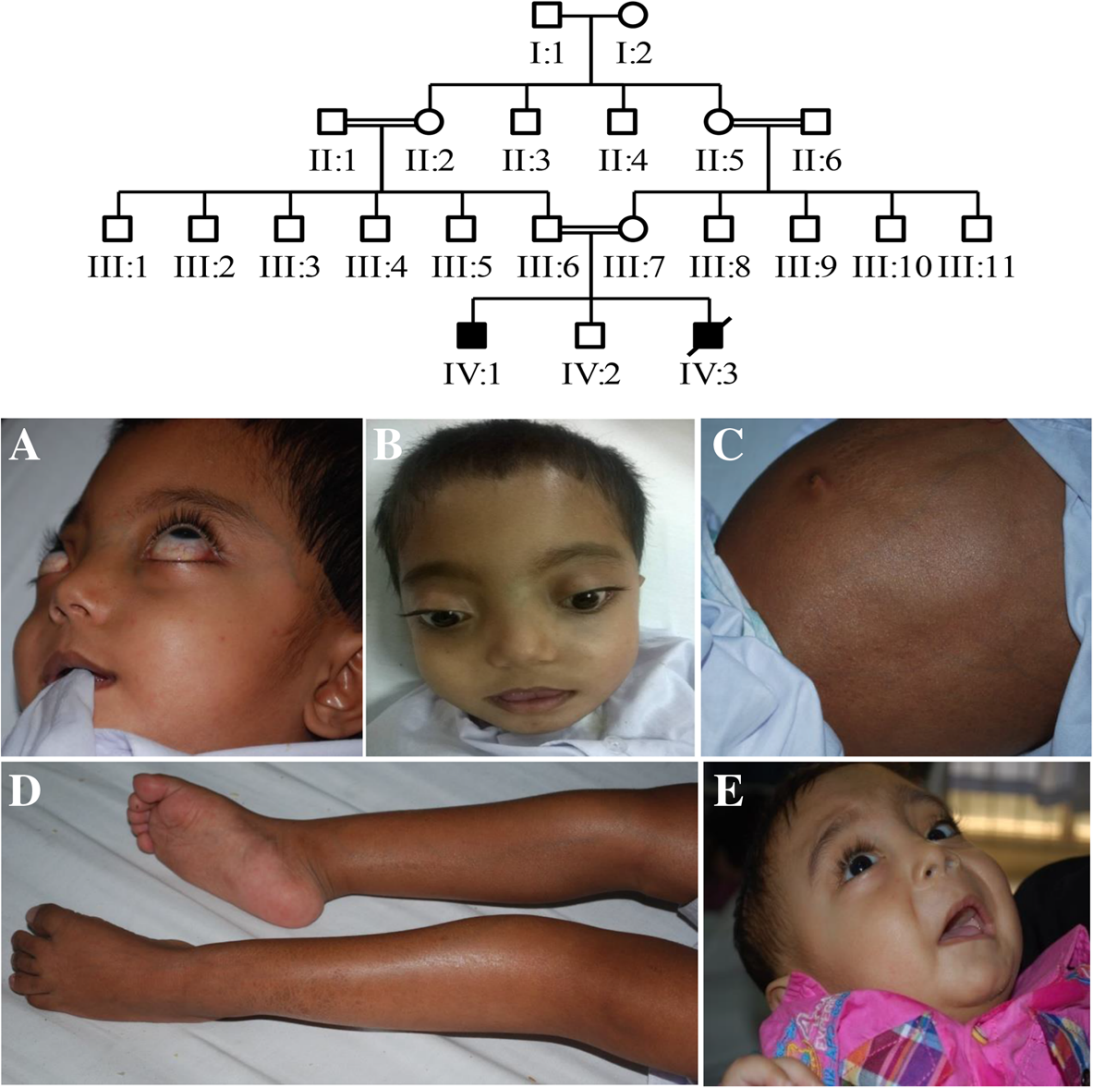
The ARO family pedigree & clinical presentation of patients. Upper panel; Pedigree of consanguineous multi-generation Pakistani family demonstrating segregation of osteopetrosis as an autosomal recessive trait. Lower panel; (**a**) Patient IV-1, a 3-year-old boy with craniofacial symptoms showing exophthalmoses (**b**) macrocephaly, frontal bossing, flat nasal bridge and hypertelorism (**c**) protuberance of abdomen due to hepatosplenomegaly (**d**) limb deformities and muscle wasting. (**e**) Patient IV-3, a 1-year-old boy with less severe skeletal features including macrocephaly, flat nasal bridge with hypertelorism and mild exophthalmos

A multi-generational consanguineous family with a history of osteopetrosis was identified from a secluded area of KPK province of Pakistan (Fig. 1). Two affected siblings, ages between 1 and 3 years, were presented with characteristic features of osteopetrosis while their parents and a sibling identified phenotypically healthy. Family members were consented to participate in the study and their blood samples were drawn. Blood samples were also collected from 100 ethnically-matched unrelated normal individuals to be used as a control for allele frequency calculation and confirmation of disease-associated mutation. Genomic DNA from peripheral blood mononuclear cells was extracted by standard phenol-chloroform-based DNA extraction procedure [13]. The study was approved by institutional ethic committee (Ethical Committee, IB&GE Islamabad, Pakistan (Ref. No. IBGE/IEC/18/01/16) and was in concordance with the Helsinki declaration.

### SNP genotyping and homozygosity mapping

DNA samples from five family members (two affected siblings, one unaffected sibling and their parents) were genotyped on the Illumina OmniExpress v1.1 BeadChip array for a total of 712,526 genetic markers. After removal of single nucleotide polymorphisms (SNPs) that were non-polymorphic or failed genotyping in at least one sample within the pedigree, 314,385 SNPs were further analyzed. We confirmed the reported familial relationships among all five genotyped samples using identity-by-descent analysis on PLINK [14]. To identify homozygous segments (>1 Mb in length) that are shared between the two affected individuals but not with any unaffected individual, homozygosity mapping was conducted using PLINK v1.07 and Homozygosity Mapper using the default settings (http://homozygositymapper.org /).

### Mutation screening

Intronic primers (see Additional file 1: Table S1) were designed to amplify exons and adjacent splice site sequences to identify any potential disease associated variant of *TCIRG1* gene. Amplified PCR products were purified using QIAquick PCR Purification Kit (Qiagen, U.K.) and subjected to sequencing by using BigDye®Terminator v3.1 cycle sequencing kit on an ABI3130 genetic analyzer (Applied Biosystems, U.S.A.). Potential disease-associated mutation was confirmed by bidirectional sequencing, allele-specific PCR and assessing hundred control samples having similar ethnic background.

### Sequence acquisition

Reference sequences of *TCIRG1* gene (NG_007878.1), coding nucleotides (NM_006019.3) and amino acids (NP_006010.2) were retrieved through NCBI database.

### Mutation analysis by allele-specific PCR

The identified deletion mutation in exon 6 of *TCIRG1* was further validated by using allele-specific primers that were designed to match wild-type sequences (see Additional file 1: Table S2). In a single reaction, one allele-specific inner primer (forward or reverse) was used in combination with two common outer primers to amplify the candidate region. Amplification was achieved in a 25 μl final reaction volume containing 200 ng genomic DNA, 1 unit of Taq DNA polymerase and 0.6 μM of each primer. Amplification was performed with an initial denaturation at 95 °C for 5 min, followed by 35 cycles of denaturation at 95 °C for 45 s, primer-specific annealing temperature for 45 s, 72 °C for 45 s and a final extension at 72 °C for 10 min. In the present study we used allele-specific inner forward primer only and amplified products were separated on 2% agarose gel and genotypes were called by visual inspection. To understand expected outcomes of allele-specific amplification against different possible genetic makeup, a key is proposed (see Additional file 1: Table S3).

### Bioinformatics analysis

To predict secondary as well as three dimensional (3D) structures of the wild-type and mutant TCIRG1 protein, Psipred and I-Tasser bioinformatics tools were used respectively [15, 16]. Protein 3D–models reliability was checked using RAMPAGE server and visualized through ViewerLite v5.0 [17]. STITCH4 database was used to predict functional protein partners [18]. Pockets/interaction sites of protein were identified using Computed Atlas of Surface Topography of proteins (CASTp) [19, 20]. Docking analysis was carried out using PatchDock server [21, 22]. The refinement of first 10 docked complexes, obtained through PatchDock, was carried out using FireDock [23, 24]. Representations (2-Dimensional) and analysis of protein-ligand interaction complexes was done using LIGPLOT [25].

## Results

### Clinical investigations

Though all typical symptoms of the disease were not manifested at the time of birth, possibility of inborn genetic defect could not be excluded. The first comprehensive clinical examination of affected individuals IV: 1 and IV: 3 (Fig. 1) was carried out during the first 5 months of their infancy. Both affected individuals’ revealed typical signs and symptoms attributed to malignant infantile osteopetrosis. Severity of the clinical manifestations was significantly varied between the two individuals. According to the information provided by physician and parents of affected individuals, at the time of first clinical evaluation, individual IV: 1 was presented with marked retardation of growth and development and his length and weight were below the 3rd percentile for age. Other physical findings included macrocephaly with head size >98th percentile for age. Frontal bossing, bilateral exophthalmos, absent pupillary light reflexes and minimum response to auditory stimuli were also the clinical observations. He also had marked hepatosplenomegaly. Patient IV: 3 has length at 25th percentile for age and weight at 50th percentile. He had almost normal skull size. There was mild hepatosplenomegaly. He was completely non-responder to visual stimuli and response to auditory stimuli was almost normal. He had flat nasal bridge with hypertelorism and mild exophthalmos.

**Fig. 2:**
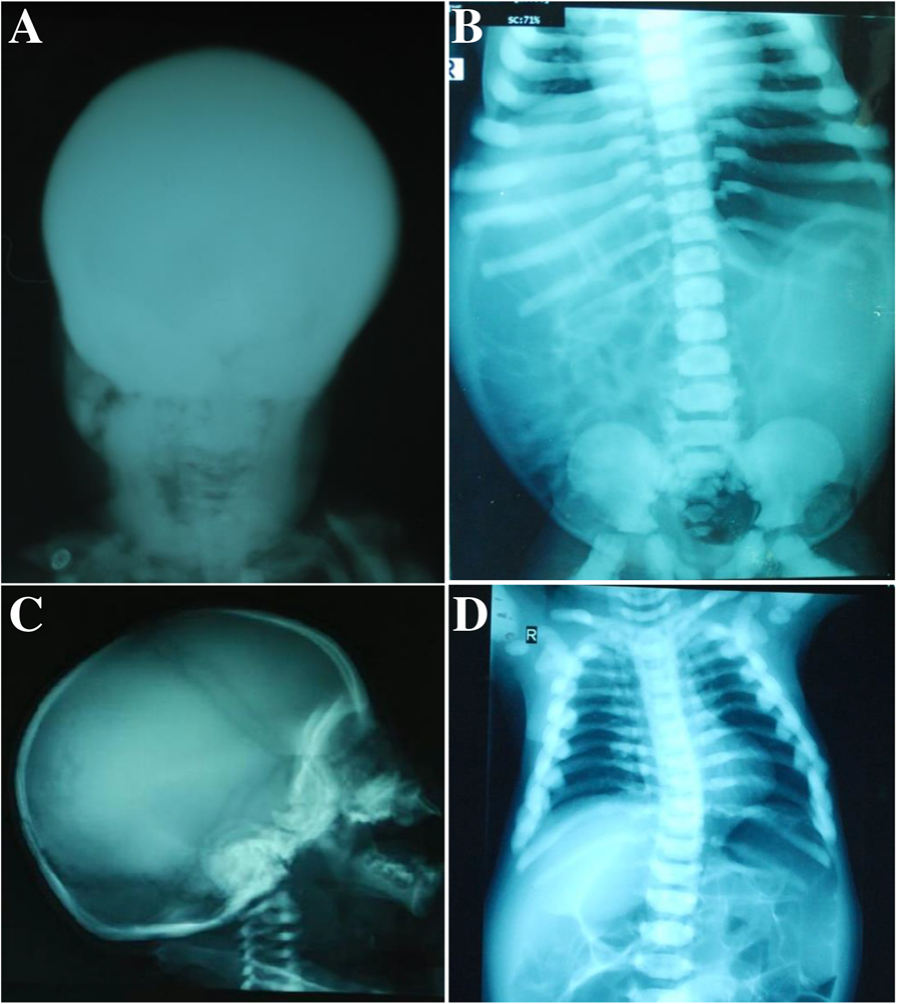
Radiological investigations. **a** Individual IV: 1; image shows diffusely thickened and sclerotic skull with increased density of visualized bones **b** Prominent diffuse increase in bone density in ribs, visualized dorosolumber spine and pelvic bones as well as widening of costochondral junctions. **c** Individual IV: 3, image shows diffuse dense calvarium with increased density of rest of the visualized bones **d** Generalized diffuse increase in bone density, mild scoliosis of dorsal spine with convexity towards left side and loss of corticomedullary differentiation

At the time of second clinical evaluation when the family was enrolled for this study, individuals IV: 1, a 3-years-old boy (Fig. 1a-d) was presented with macrocephaly measuring occipital frontal circumference (OFC) 58 CM and prominent frontal bossing. Eye features presented severe exophthalmos with exposure keratitis leading to gradual visual loss of both eyes. Other craniofacial abnormalities were including the flattened nasal bridge and misaligned teeth with dental caries. Abdomen was grossly distended with everted umbilicus and tense with massive hepatosplenomegaly. He also had marked muscle wasting, more obvious in the lower limbs, with multiple bruises on the shins. Radiological findings revealed abnormalities with diffusely thickened and sclerotic skull along with an increased density of the rest of visualized bone (Fig. 2a). The chest x-rays also support the osteoporotic features as diffuse increase in bone density in ribs, visualized dorosolumber spine and pelvic bones is prominent. Widening of costochondral junctions is also present (Fig 2b).

The individuals IV: 3, a 1-year-old boy (Fig. 1e), a diagnosed case of osteopetrosis, had mild/borderline macrocephaly measuring occipital frontal circumference (OFC) 50 CM with patent anterior fontanelle. He had flat nasal bridge with hypertelorism and mild exophthalmos. Radiological investigations (X-rays) are suggestive of osteopetrotic phenotype as evidenced by abnormally dense culvarium along with increased density of rest of the visualized bones (Fig. 2c), generalized diffuse increase in bone density, widening of costochondral junctions along with loss of corticomedullary differentiation and undertubularization seen in visualized humeri (Fig. 2d). Some disease associated features like teeth anomalies, progressive deafness and frontal bossing could not significantly be concluded in this patient due to an early death at the age of 1 year as a result of pneumonia leading to congestive heart failure.

### SNP genotyping & mutation analysis of *TCIRG1* gene

SNP-based genotyping and homozygosity mapping revealed multiple regions of shared homozygosity (among two affected individuals only) on different chromosomes. While comparing identified homozygosity regions with previously reported loci, we came up with a 3.97 Mb region on chromosome 11 harboring the *TCIRG1* gene that has been previously reported to be mutated in autosomal recessive osteopetrosis (see Additional file 1: Figure S1, Figure S2, Table S4, Table S5).

**Fig. 3:**
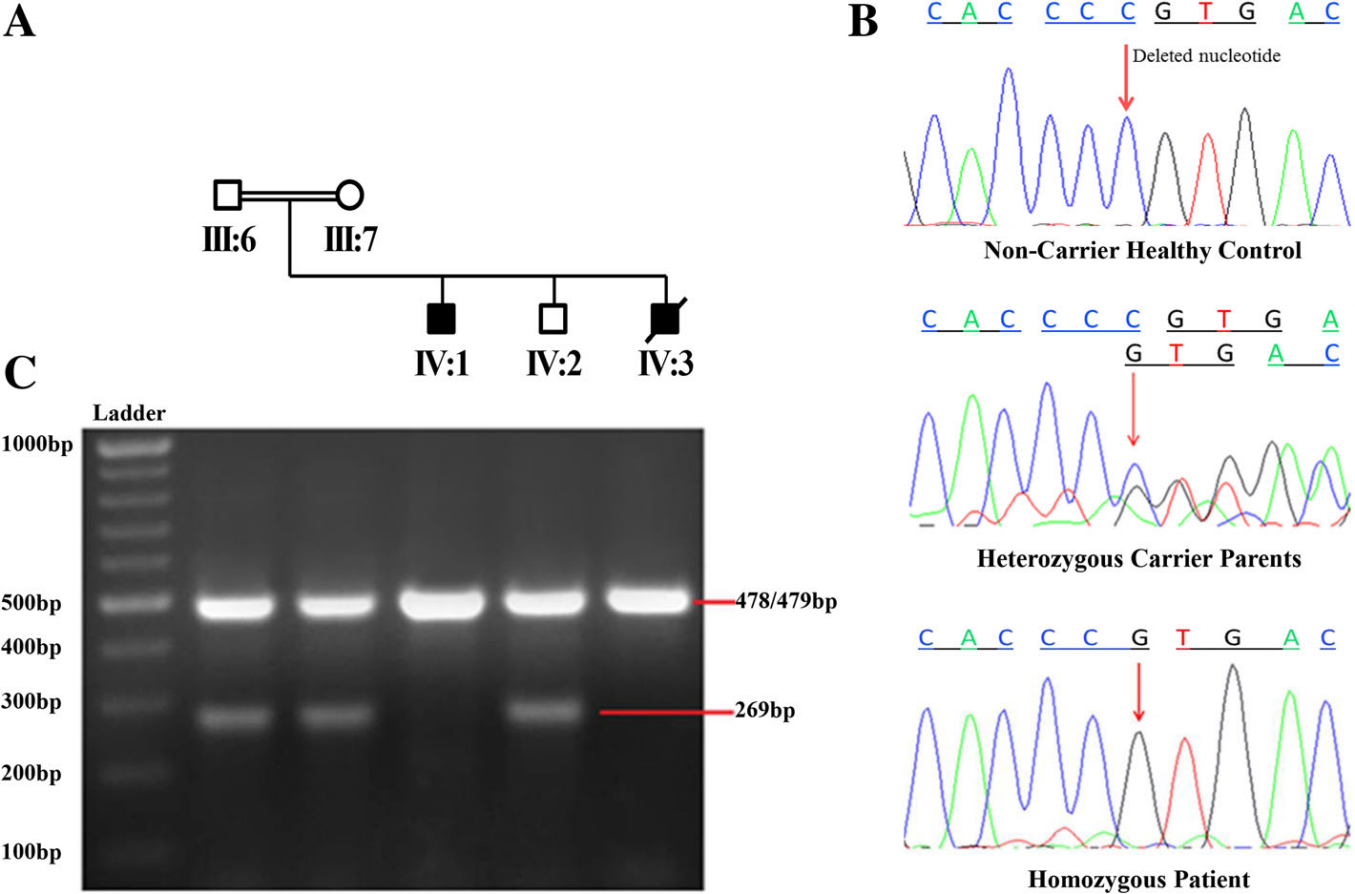
Mutation analysis. **a** Branch of the affected family subjected to mutation analysis. **b** Sanger sequencing of exon 6 of the *TCIRG1* gene illustrating c.624delC (p.P208PfsX1) variation. The affected individuals are homozygous for this deletion, whereas phenotypically normal parents are found heterozygous having normal as well as mutant allele. Site of variation is indicated by arrow. **c** Allele-specific amplification is also supporting the homozygous deletion mutation as allele-specific band of 269 bp is present in carrier individuals (III-6, III-7, IV-2) while absent in affected subjects (IV-1 & IV-3)

Sanger sequencing of available family member (Fig 3a) revealed a novel homozygous cytosine deletion in exon 6 of the *TCIRG1* (c.624delC) in both affected individuals. Both parents and a sibling were found heterozygous for this deletion (Fig. 3b). PCR amplification with common outer primers, and a combination of outer and an allele-specific inner forward primer further validated the Sanger sequencing results. The fragments of sizes 478 bp/479 bp and 269 bp were observed in heterozygous carriers while only one fragment of 478 bp was observed in homozygous patients. The differentiation between 478 bp and 479 bp DNA fragments was not possible due to the resolution limitation with 2% agarose gel, that’s why they were observed as a single band (Fig. 3c). The presence of allele-specific band (269 bp) in carrier individuals and its absence in affected subjects confirms homozygous deletion of a nucleotide. In the present study allele-specific inner reverse primer was not used (see Additional file 1: Table S3).

The identified mutation was not observed in public databases or in any of the 100 control samples. The results were therefore, consistent with the recessive mode of disease segregation in this family.

### Comparative protein modeling and in silico analysis

**Fig. 4:**
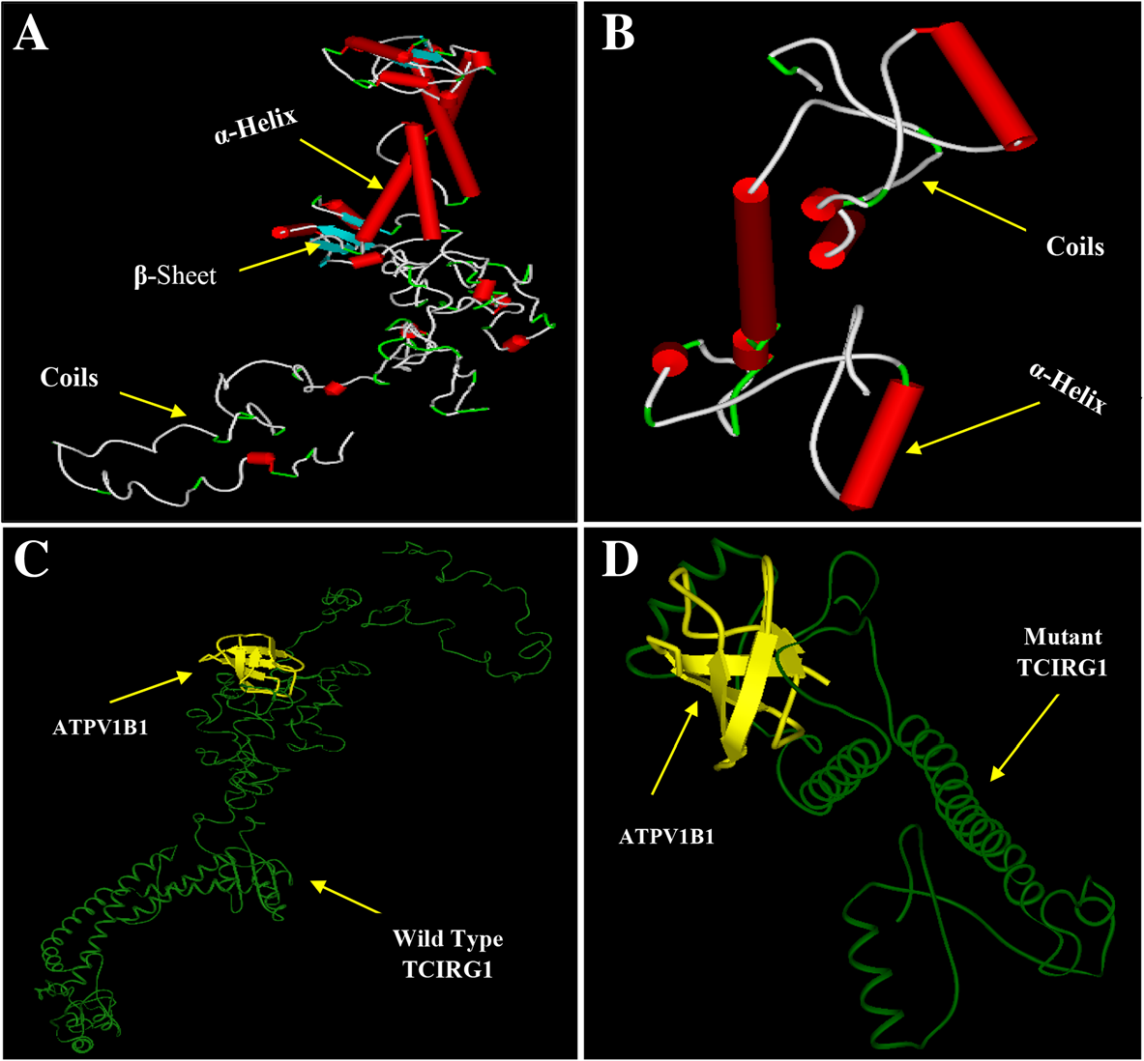
3D–Models. **a** Normal TCIRG1 **b** Mutant TCIRG1 (p.P208PfsX1)**.** Docking complex visualization: **c** TCIRG1 vs ATPV1B1 **d** Mutant TCIRG1 vs ATPV1B1. Receptor protein has been shown in solid ribbon display style with green color; ligand has been shown in secondary type with yellow color

**Fig. 5:**
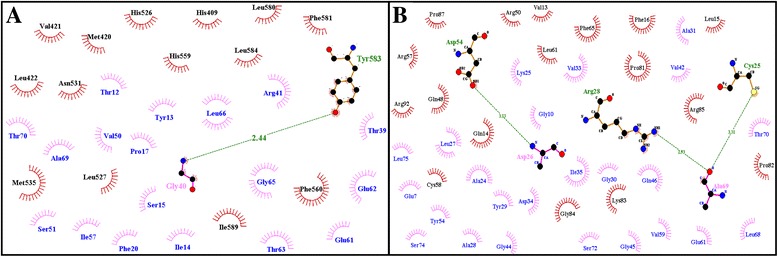
2D–DimPlot representation of docking interaction. **a** Normal TCIRG1 with ATPV1B1. **b** Mutant TCIRG1 with ATPV1B1. Receptor and ligand residues involved in hydrophobic interactions are represented by brick red and pink spoke arcs () respectively. Hydrogen bonding is shown by green dotted lines (). Receptor residues involved in hydrogen bonding are labeled in olive green color. Ligand residues involved in hydrogen bonding are labeled in pink color

Comparative analysis of secondary structures features of the wild and mutant types unveiled that the normal protein comprises of 27 helices, 12 strands and 39 coils. Whereas, a single nucleotide deletion resulted in gross changes in the 3D–protein structure as determined by decrease in the total numbers of helices, strands and coils to 6, 3, and 10, respectively. The impact of the deletion has been illustrated in a graphical comparison of secondary structure features of both, the wild-type and mutant proteins (see Additional file 1: Figure S3). Likewise, the differences in number of helices, beta sheets and coils produced as a result of mutation, have been demonstrated in 3D–models of wild- type and mutant respectively (Fig. 4a-b). Using STITCH4 database, we identified a ligand protein ATPase V1 subunit B1 for having high level of interaction affinity to receptor protein. ATPase V1B1 is also responsible for acidifying a variety of intracellular compartments in eukaryotic cells. Protein docking study explains the protein-protein complexes of small ATPV1B1 protein molecule with macromolecules i.e. wild-type and mutant a3 subunits respectively (Fig. 4c-d). Docking complexes are formed due to the hydrogen bonding and hydrophobic interactions between molecules at specific interaction sites/pockets. Part A & B of the Fig. 5 demonstrates the possible docking interaction of ligand protein with wild-type and mutant a3 subunit respectively. Different amino acid residues from receptor and ligand proteins are involved in hydrogen bonding and hydrophobic interactions. These interactions showed that amino acid residue Tyr583 from wild-type a3 subunit is engaged in hydrogen bonding with Gly40 residue of the ligand protein for docking complex (Fig 5a). Whereas, in case of mutated a3 subunit, the residues Cys25, Arg28 and Asp54 are involved in hydrogen bonding with Asp26 and Ala69 residues of ligand protein (Fig 5b). Amino acid residues involved in hydrogen bonding and hydrophobic interactions are summarized in Table 1. Significant dislocation and change in hydrogen bonding pattern, hydrophobic interaction sites and amino acid residues involved in receptor-ligand complexes, clearly implicate the pathogenic nature of deletion mutation.

**Table 1:** Docking interactions: amino acid residues involved in hydrogen bonding and hydrophobic interactions

Receptor-Ligand	Hydrogen Bonding		Hydrophobic Interactions		Figure
	Ligand Residues	Receptor Residues	Ligand Residues	Receptor Residues	
TCIRG1- ATPV1B1	Gly40	Tyr583	Thr12 Tyr13 Ile14 Ser15 Pro17 Phe20 Thr39 Arg41 Val50 Ser51 Ile57 Glu61 Glu62 Thr63 Gly65 Leu66 Ala69 Thr70	His409 Met420 Val421 Leu422 His526 Leu527 Asn531 Met535 His559 Phe560 Leu580 Ohe581 Leu584 Ile589	Fig. 5a
Mutant TCIRG1- ATPV1B1	Asp2Ala69	Cys2Arg28Asp54	Glu7 Gly10 Ala24 Lys25 Leu27 Ala28 Try29 Gly30 Ala31 Val33 Asp34 Ile35 Val42 Gly44 Gly45 Gln46 Tyr54 Val59 Glu61 Leu68 Thr70 Ser72 Ser74 Leu75	Val13 Gln14 Leu15 Phe16 Gln48 Arg50 Arg57 Cys58 Leu61 Phe65 Pro81 Pro82 Lys83 Gly84 Arg85 Pro87 Arg92	Fig. 5b

## Discussion

Bone resorption process requires a constant secretion of acid (H^+^) into the resorption lacunae mediated by V-ATPase proton pump [10]. The V-ATPase proton pump is multi-subunits membrane complex consisting of two main domains; a cytosolic hydrolytic domain (V1) that mediates ATP hydrolysis and a transmembrane proton translocation domain (V0) that facilitates extracellular acidification of organelles [10, 26]. Mutations, deletions or genes knockdown of different subunits of the V-ATPase complex have been described to be implicated in impaired osteoclastic bone resorption, leading to severe osteopetrosis [27]. Approximately 50% of the patients with recessive infantile malignant osteopetrosis have mutations to a3 subunit of V-ATPase protein complex, encoded by *TCIRG1* gene [28]. To date, over hundred pathogenic variations of *TCIRG1* gene including twenty small deletion mutations have been reported to be associated with impaired a3 subunit function, hence infantile malignant osteopetrosis (HGMD; www.hgmd.org).

In the present study we identified a homozygous deletion mutation (c. 624delC) in exon 6 of the *TCIRG1* gene and to the best of our knowledge it has not been reported before. The deletion led to a frame shift that created a stop codon at position downstream next to the point of deletion. Hence, resulted premature truncated protein consists of 208 aa instead of normal 830 aa (p.P208PfsX1). In other words there is a loss of 622 aa at C-terminal domain of a3 subunit. The occurrence of deletion in homozygous state in all affected subjects, heterozygous state in both carrier parents and absence in a screen of 100 ethnically matched healthy controls confirms its association with infantile malignant osteopetrosis in Pakistani family.

In silico characterization of the deletion mutation revealed significant consequences on the secondary structure features of mutant type protein (see Additional file 1: Figure S3). Premature termination not only led to an abnormal shorter protein of 208 aa but also resulted in atypically reduced numbers of structural motives like alpha helices, strands and coils. Structural changes might have resulted in amino acid re-arrangement and conformational changes so that the receptor-ligand interaction trend of mutant a3 subunit is predicted totally different from that of wild-type a3 subunits (Fig. 4c-d). The 2-dimensional protein-ligand interaction complexes predicted hydrogen bonding between Tyr583 of wild-type receptor and Gly40 of ligand molecule ATPV1B1. Whereas, in case of mutant protein, the residues Cys25, Arg28 and Asp54 are involved in hydrogen bonding with Asp26 and Ala69 residues of protein-ligand (Fig. 5a-b). Significantly dislocated interaction sites and amino acid residues have also disturbed the hydrophobic interactions that are equally important in receptor-ligand interactions. Location of interaction sites and number of interactive pockets, involvement of different amino acid residues as well as hydrogen bonding pattern are in Table 1.

Topological studies propose a two-domain structure for “a3 subunit” with an N-terminal cytosolic domain necessary to target V-ATPase to the plasma membrane of osteoclasts, and a membrane integral C-terminal domain that make structural frame work of V0 domain and affects the coupling of proton transport and ATP hydrolysis [10, 29]. In the present study, loss of 622 aa at C-terminal of a3 subunit strongly suggests impaired proton coupling, transportation and ATP hydrolysis even if it is supposed that the N-terminal with 208 aa targets V-ATPase to the plasma membrane of osteoclasts. It can also be hypothesized that partially translated a3 subunit may have been degraded by “nonsense mediated mRNA decay” [26], resulting in a complete loss of a3 subunit. In either situation, there is a strong possibility of V-ATPase complex assembly disruption, leading to clinical phenotypes of osteopetrosis.

## Conclusions

In summary, the mutant a3 isoform might have caused V-ATPase complex assembly to be disrupted, resulted in osteoclast failure to maintain extracellular acidification and hence defective bone resorption attributed to infantile malignant osteopetrosis. Our results, supported by SNP genotyping, Sanger sequencing, allele-specific PCR and bioinformatics have not only expanded the spectrum of *TCIRG1* pathogenic variations but also the body of evidences supporting the role of a3 isoform in osteopetrosis. Moreover, our findings may be supportive for prenatal genetic screening and early diagnosis of osteopetrosis.

## Additional file

Additional file 1: Table S1. Intronic primers used to amplify coding exons of TCIRG1 gene; **Table S2.** Allele-specific PCR primers for c.624delC mutation in TCIRG1 gene; **Table S3.** Expected outcomes of allele-specific amplification; **Table S4.** Location of homozygous segments on genome shared by cases; **Table S5.** Known loci for osteopetrosis; **Figure S1.** Linked loci extracted by using Homozygosity mapper; **Figure S2.** Linked region at Chr11, showing TCIRG1 as candidate gene; **Figure S3.** Comparison of predicted secondary structure features for wild-type and mutant TCIRG1 protein. (DOCX 724 kb)

## Abbreviations

ADO: Autosomal dominant osteopetrosis
ARO: Autosomal recessive osteopetrosis
OFC: Occipital frontal circumference
OPT: Osteopetrosis
OPTB1: Malignant infantile osteopetrosis 1
