# Supplementary material for: Identification and in silico characterization of a novel p.P208PfsX1 mutation in V-ATPase a3 subunit associated with autosomal recessive osteopetrosis in a Pakistani family
Source: BMC Med Genet. 2017 Dec 13;18:148. doi: 10.1186/s12881-017-0506-4 (PMC5729456; doi:10.1186/s12881-017-0506-4)
Supplement: Additional file 1: Table S1. — Intronic primers used to amplify coding exons of TCIRG1 gene; Table S2. Allele-specific PCR primers for c.624delC mutation in TCIRG1 gene; Table S3. Expected outcomes of allele-specific amplification; Table S4. Location of homozygous segments on genome shared by cases; Table S5. Known loci for osteopetrosis; Figure S1. Linked loci extracted by using Homozygosity mapper; Figure S2. Linked region at Chr11, showing TCIRG1 as candidate gene; Figure S3. Comparison of predicted secondary structure features for wild-type and mutant TCIRG1 protein. (DOCX 724 kb) [file 12881_2017_506_MOESM1_ESM.docx]

**SUPPLEMENTARY MATERIAL**

**Table S1.** Intronic primers used to amplify coding exons of *TCIRG1* gene.

| **Gene** | **Primer ID** | **Primer Sequence (5’-3’)** | **Ta °C** | **Product (bp)** |
| --- | --- | --- | --- | --- |
| TCIRG1 | TCIRG1-E2/3_F | CAGTGAGTGAAGGTGCACAGG | 60 | 669 |
|  | TCIRG1-E2/3_R | TGCCTGGAATGTAGGCCTGG |  |  |
|  | TCIRG1-E4/5_F | CCTCAACTGTTGGAGACAACCTC | 58 | 535 |
|  | TCIRG1-E4/5_R | ACAAGGAGTCGGAGCTCAGC |  |  |
|  | TCIRG1-E6/7_F | TGCCCAATTGCCCGATTGC | 58 | 479 |
|  | TCIRG1-E6/7_R | TGGGGAGGAGTCACGATAGG |  |  |
|  | TCIRG1-E8/9_F | CAGACTCAGACTCTCGTAGC | 58 | 667 |
|  | TCIRG1-E8/9_R | CTGGAAGTGAGGCAGAAACG |  |  |
|  | TCIRG1-E10_F | GCTGATCATCTCACGTCAGAG | 55 | 464 |
|  | TCIRG1-E10_R | CCTCACACTGGCTGCAGAGC |  |  |
|  | TCIRG1-E11-13_F | GGCAGATGCTGGTGTGTTCG | 60 | 674 |
|  | TCIRG1-E11-13_R | CAGGACGGCTGAACCGAGG |  |  |
|  | TCIRG1-E14/15_F | GGACTTCCTGGCAGTGATGG | 58 | 550 |
|  | TCIRG1-E14/15_R | TCCCAGTGGCCCTGTGACC |  |  |
|  | TCIRG1-E16-17_F | TTGCAGGTGTGCACAGCAGG | 62 | 669 |
|  | TCIRG1-E16-17_R | CAGCCGTCGGTGGCCAGG |  |  |
|  | TCIRG1-E18_F | GCCTGGATGATGAAGAGGAG | 62 | 307 |
|  | TCIRG1-E18_R | AACTGAGGCCCAGAGAGAAG |  |  |
|  | TCIRG1-E19/20_F | CTGGCAGGCACCCACTTGC | 60 | 472 |
|  | TCIRG1-E19/20_R | GACGAGACATCACTGCCAGG |  |  |

F= forward primer; R = reverse primer; Ta = optimal annealing temperature; bp = base pair

Highlighted green: primers used to amplify exon 6 of the TCIRG1.

Note: First exon of the *TCIRG1* gene is a non-coding exon.

**Table S2.** Allele-specific PCR primers for c.624delC mutation in *TCIRG1* gene

| **Gene** | **Primer ID** | **Description** | **Primer Sequence (5’-3’)** | **Ta °C** |
| --- | --- | --- | --- | --- |
| TCIRG1 | E6/7_F | Common outer forward primer | TGCCCAATTGCCCGATTGC | 61 |
|  | E6/7_R | Common outer reverse primer | TGGGGAGGAGTCACGATAGG |  |
|  | Allele-specific _F | c.624delC specific inner forward primer | GCAGCCGCTGGAGCACCCC |  |
|  | Allele-specific _ R | c.624delC specific inner reverse primer | ccagctgctcacCGTCACG |  |

F= forward primer; R = reverse primer; Ta = optimal annealing temperature; bp = base pair

**Table S3.** Expected outcomes of allele-specific amplification

| **Genetic status** | **Common outer primers product size (bp)** | **Allele-specific inner forward + Outer reverse primers product size (bp)** | **Allele-specific inner reverse + Outer forward primers product size (bp)** |
| --- | --- | --- | --- |
| Normal individual | 479 | 269 | 247 |
| Carrier individual | 478 + 479 | 269 | 247 |
| Homozygous patient | 478 | - | 246 |

**Note:**  In a single reaction, one allele-specific inner primer (either forward or reverse) can be used in combination with both outer primers to amplify the candidate region.

In the present study, both allele-specific_ F & R inner primers were designed to match wild type sequences for the validation of deletion mutation. However, we used allele-specific inner forward primer only and amplified products were separated on 2% agarose gel. The fragments of sizes 478bp/479bp and 269bp were observed in heterozygous carriers while only one fragment of 478bp was observed in homozygous patients. The differentiation between 478bp and 479bp DNA fragments was not possible due to the resolution limitation with 2% agarose gel, that’s why they were observed as a single band (Fig. 3C). The presence of allele-specific band (269bp) in healthy and carrier individuals and its absence in affected subjects confirms homozygous deletion of a nucleotide.

On the other hand, allele-specific inner reverse primer was not used due to two reasons; (1) Patients, despite having homozygous c.624delC mutation, possess DNA sequence complementary to the reverse primer. This is due to the presence of a short string of cytidine (5’-CCCC-3’) at the site of deletion. Hence, 3’ end cytidine deletion (c.624delC) is replaced with upstream cytidine due to frame shift and making the resulting sequence complementary to the inner reverse primer. In such situation an allele-specific fragment of 247bp and 246bp will be observed in phenotypically normal individuals and patients respectively; however, resolution of fragments with 1bp difference demands high resolution gel electrophoresis. (2) The purpose of allele-specific amplification for results validation is already achieved with inner forward primer.


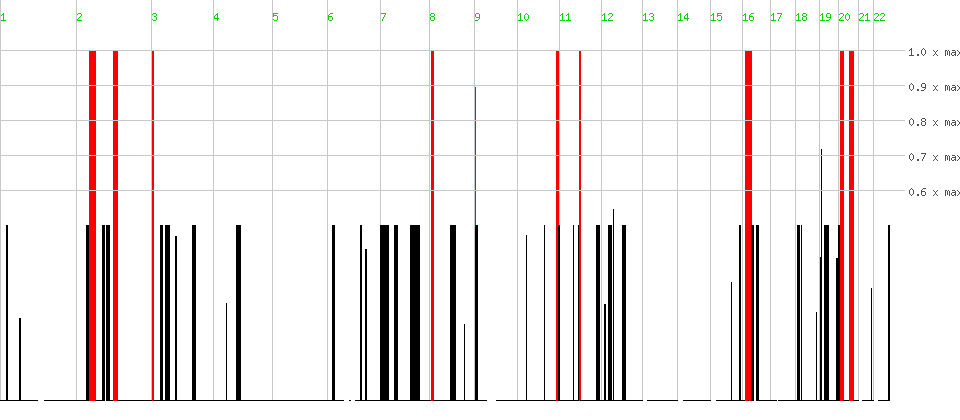


**Chr 11 (q13.1-q13.3)**

**Figure S1.** Linked loci extracted using Homozygosity mapper

**Table S4.** Location of homozygous segments on genome shared by cases

| CHR | SNP1 | SNP2 | BP1 | BP2 | KB | Genomic location |
| --- | --- | --- | --- | --- | --- | --- |
| 2 | rs4278972 | rs6713914 | 42095441 | 59523041 | 17427.6 | Chr2(p21-16.1) |
| 2 | rs272118 | rs6710001 | 121094955 | 131537733 | 10442.8 | Chr2(q14.2-q21.1) |
| 3 | rs12715157 | rs9823228 | 3006381 | 5564422 | 2558.0 | Chr3(p26.2-p26.1) |
| 3 | rs9845829 | rs4130991 | 78337174 | 79469022 | 1131.9 | Chr3(p12.3) |
| 8 | rs4831486 | rs573980 | 6655391 | 13721994 | 7066.6 | Chr8(p23.1-p22) |
| 10 | rs951631 | rs7912924 | 125285875 | 130939211 | 5653.3 | Chr10(q26.13-q26.3) |
| **11** | **rs470763** | **rs2305508** | **64584231** | **68549546** | **3965.3** | **Chr11(q13.1-q13.3)** |
| 12 | rs4254134 | rs12372413 | 40196809 | 41291402 | 1094.6 | Chr12(q12) |
| 16 | rs1103909 | rs11074725 | 10404410 | 18920881 | 8516.5 | Chr16(p13.2-p12.3) |
| 20 | rs1591640 | rs6076792 | 4034197 | 5223014 | 1188.8 | Chr20(p13-p12.3) |
| 20 | rs1555141 | rs1035179 | 5782559 | 15538827 | 9756.3 | Chr20(p12.3-p12.1) |
| 20 | rs2425047 | rs852353 | 33871675 | 46833654 | 12962.0 | Chr20(q11.22-q13.13) |

**Table S5.** Known loci for osteopetrosis

| **Type** | [**OMIM**](https://en.wikipedia.org/wiki/OMIM) | **Gene** | **Genomic location** |
| --- | --- | --- | --- |
| OPTA1 | [607634](https://omim.org/entry/607634) | *LRP5* receptor | *11q13.2* |
| OPTA2 | 166600 | *CLCN7* chloride channel | *16p13.3* |
| OPTB1 | 259700 | *TCIRG1* ATPase | *11q13.2* |
| OPTB2 | 259710 | *TNFSF11 or RANKL* | *13q14.11* |
| OPTB3 | 259730 | *CA2* (renal tubular acidosis) | *8q12.2* |
| OPTB4 | 611490 | *CLCN7* chloride channel | *16p13.3* |
| OPTB5 | 259720 | [*OSTM1*](https://en.wikipedia.org/wiki/OSTM1) ubiquitin ligase | *6q21* |
| OPTB6 | 611497 | *PLEKHM1* adapter protein | *17q21.1* |
| OPTB7 | 612301 | [*TNFRSF11A*](https://en.wikipedia.org/wiki/TNFRSF11A) (RANK receptor) | *18q21.33* |
| OPTB8 | *614780* | *SORTING NEXIN 10; SNX10* | *7p15.2* |


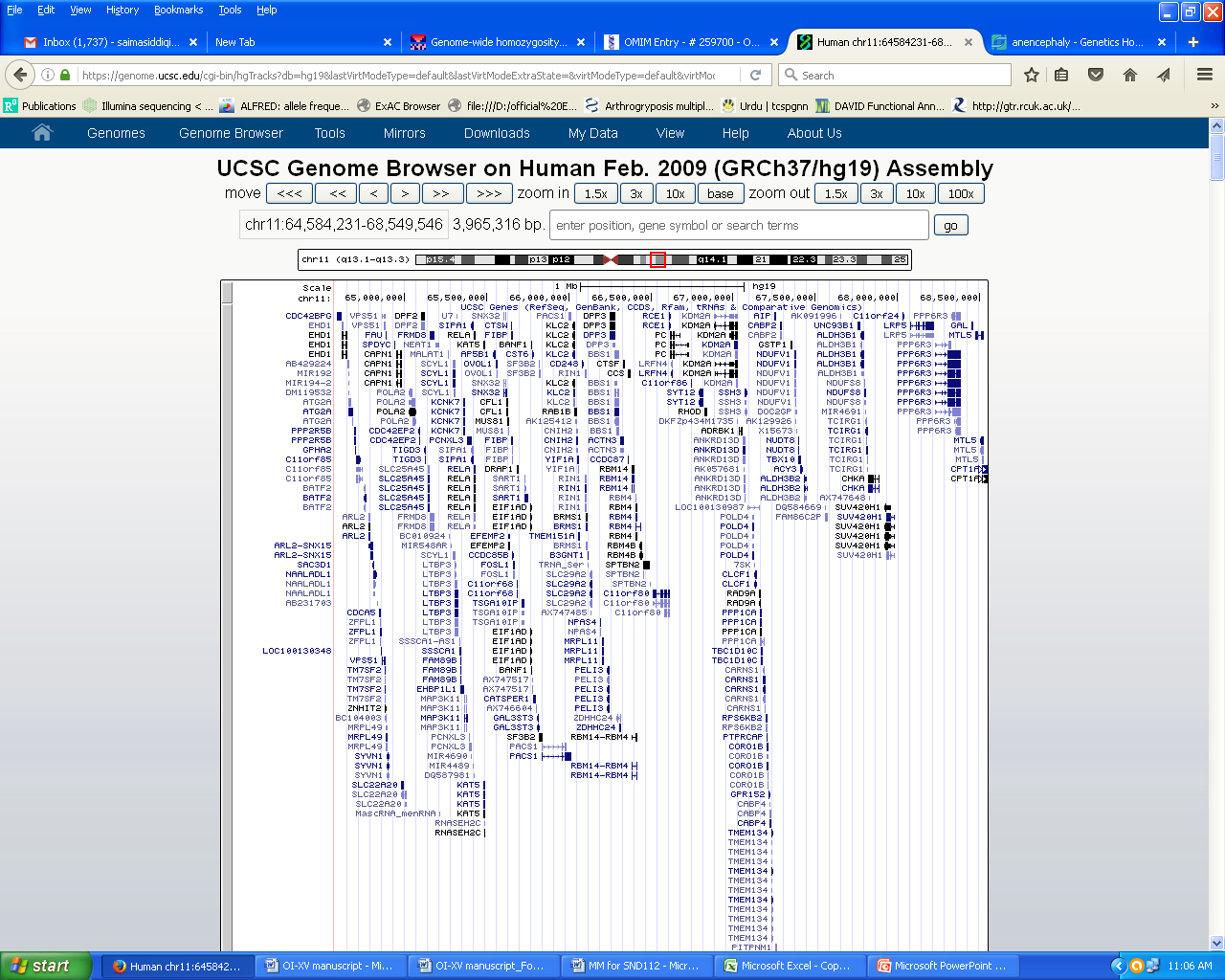


**Figure S2.** Linked region at Chr11, showing TCIRG1 as candidate gene.


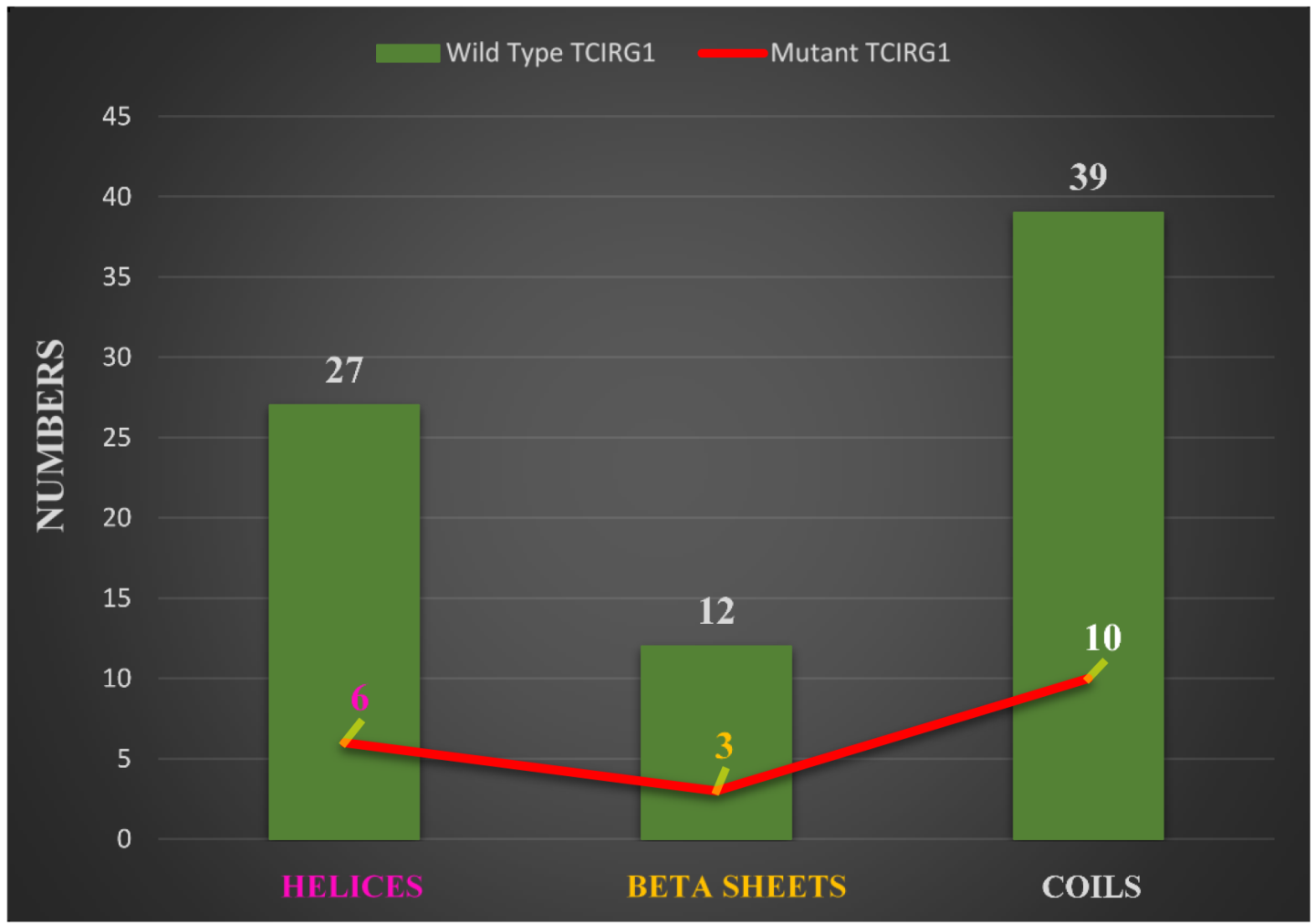


**Figure S3. Comparison of predicted secondary structure features for wild-type and mutant TCIRG1 protein**. PsiPred results**:** secondary structure analysis showed that normal protein is comprised of 27 helices, 12 strands and 39 coils whereas only 6 helices, 3 strands and 10 coils motives are present in mutated form.
